# Identification of estrogen responsive genes using esophageal squamous cell carcinoma (ESCC) as a model

**DOI:** 10.1186/1752-0509-6-135

**Published:** 2012-10-26

**Authors:** Magbubah Essack, Cameron Ross MacPherson, Sebastian Schmeier, Vladimir B Bajic

**Affiliations:** 1 Computational Bioscience Research Center (CBRC), King Abdullah University of Science and Technology (KAUST), Thuwal, Kingdom of Saudi Arabia

## Abstract

**Background:**

Estrogen therapy has positively impact the treatment of several cancers, such as prostate, lung and breast cancers. Moreover, several groups have reported the importance of estrogen induced gene regulation in esophageal cancer (EC). This suggests that there could be a potential for estrogen therapy for EC. The efficient design of estrogen therapies requires as complete as possible list of genes responsive to estrogen. Our study develops a systems biology methodology using esophageal squamous cell carcinoma (ESCC) as a model to identify estrogen responsive genes. These genes, on the other hand, could be affected by estrogen therapy in ESCC.

**Results:**

Based on different sources of information we identified 418 genes implicated in ESCC. Putative estrogen responsive elements (EREs) mapped to the promoter region of the ESCC genes were used to initially identify candidate estrogen responsive genes. EREs mapped to the promoter sequence of 30.62% (128/418) of ESCC genes of which 43.75% (56/128) are known to be estrogen responsive, while 56.25% (72/128) are new candidate estrogen responsive genes. EREs did not map to 290 ESCC genes. Of these 290 genes, 50.34% (146/290) are known to be estrogen responsive. By analyzing transcription factor binding sites (TFBSs) in the promoters of the 202 (56+146) known estrogen responsive ESCC genes under study, we found that their regulatory potential may be characterized by 44 significantly over-represented co-localized TFBSs (cTFBSs). We were able to map these cTFBSs to promoters of 32 of the 72 new candidate estrogen responsive ESCC genes, thereby increasing confidence that these 32 ESCC genes are responsive to estrogen since their promoters contain both: a/mapped EREs, and b/at least four cTFBSs characteristic of ESCC genes that are responsive to estrogen. Recent publications confirm that 47% (15/32) of these 32 predicted genes are indeed responsive to estrogen.

**Conclusion:**

To the best of our knowledge our study is the first to use a cancer disease model as the framework to identify hormone responsive genes. Although we used ESCC as the disease model and estrogen as the hormone, the methodology can be extended analogously to other diseases as the model and other hormones. We believe that our results provide useful information for those interested in genes responsive to hormones and in the design of hormone-based therapies.

## Background

Esophageal cancer (EC) comprises of heterogeneous groups of tumors that differ in pathogenesis and etiological and pathological features. EC ranks among the ten most frequent cancers worldwide with regionally dependent incidence rates and histological subtypes [1,2]. Statistics indicate that EC mortality rates are very similar to incidence rates due to the relatively late stage of diagnosis, the poor efficacy of treatment [2], and the poor prognosis of EC result in a five year survival rate of 5-20% [3]. The most recurrent histological subtype is esophageal squamous cell carcinoma (ESCC), followed by adenocarcinoma (ADC) [4]. ESCC has a worse prognosis than ADC due to the primary ESCC tumor being in contact with the tracheobronchial tree in 75% of cases, while ADC is found below the tracheal bifurcation in 94% of cases [5].

The striking 3-4:1 male predominance of ESCC was previously ascribed to the different patterns of smoking and drinking between males and females. However, more recently Bodelon et al. reported that current users of estrogen and progestin therapy show reduced risk of ESCC [6]. Previous research supports this finding as several groups have reported estrogen induced gene regulation in esophageal squamous cell carcinoma (ESCC) and Barrett’s esophageal adenocarcinoma (BEAC) [7-12]. Moreover, Wang et al. specifically demonstrated that serum level of estradiol of ESCC patients from the high risk areas were significantly lower compared to healthy controls from both high and low risk areas and suggested the use of estrogen analogues as promising targets for the prevention and treatment of ESCC [13]. Additionally, published scientific data shows that estrogen induces an inhibitory effect on esophageal carcinoma by activating the estrogen receptor (ER) [7-9]. The activated ER functions as a transcription factor that binds to a specific TFBS known as the estrogen response element (ERE) [14,15]. There are two ER subtypes, ERα and ERβ, that are encoded on human chromosomes 6q25.1 [16] and chromosome 14q22-24 [17], respectively. Both ERα and ERβ bind to the same EREs, but ERα does so with an approximately twofold higher affinity [18]. Additionally, ERβ is known to bind to ERα suppressing ERα function [19,20]. The inverse biological effect associated with the two ER subtypes has been confirmed to exist in ESCC [7]. This collation of research findings suggests that the estrogen based therapies which have improved survival rates of cancer types such as: prostate cancer [21], lung cancer [22], brain and spinal cord tumors [23], and breast cancer [24], may also improve the outcome of ESCC.

Our current study aims at identifying estrogen responsive genes by using ESCC as a model. Potentially, such genes could be affected by estrogen. We propose a methodology that provides insight into the underlying regulation of estrogen responsive ESCC genes. We mapped EREs to the promoters of 418 ESCC genes using the Dragon ERE Finder version 6.0 (http://apps.sanbi.ac.za/ere/index.php) [25]. The 418 ESCC genes were divided into two groups: 1) genes whose promoters contain predicted EREs, and 2) genes lacking predicted EREs. These two gene groups were further divided into those known to be experimentally confirmed as estrogen responsive and those that are not. To accomplish this the 418 ESCC genes were cross checked against two databases housing estrogen responsive genes, namely KBERG [26] and ERtargetDB [27] databases. At the time of analysis the KBERG database contained 1516 experimentally confirmed estrogen-responsive genes. The ERTargetDB database contained: (a) 40 genes with 48 experimentally verified ERE direct binding sites and 11 experimentally verified ERE tethering sites; (b) 42 genes identified via ChIP-on-chip assay for estrogen binding and (c) 355 genes from gene expression microarrays, all of which were included in this study. However, this study excludes the 2659 computationally predicted estrogen responsive genes included the ERTargetDB, database. Thus this study defines estrogen responsive genes as genes that can be modulated by an external estrogen source.

We classified the 418 ESCC genes into the following four categories (Table 1):

C1/ESCC genes with predicted EREs in their promoters and known as estrogen responsive,

C2/ESCC genes with predicted EREs in their promoters but not known as estrogen responsive,

C3/ESCC genes having no predicted EREs in their promoters, but known as estrogen responsive,

C4/ESCC genes having no predicted EREs in their promoters and not known as estrogen responsive.

**Table 1:** ESCC genes categorized based on ERE predictions and experimental evidence of estrogen responsiveness

**Category**	**ERE predictions**	**Experimental evidence**	**No. of genes**
1	yes	yes	56
2	yes	no	72
3	no	yes	146
4	no	no	144

We used these categories to develop a methodology for the identification of co-localized TFBSs (cTFBSs) that characterize the promoters of the known estrogen responsive gene sets (class (C1 and C3)) as opposed to the background set (class C4). These significant cTFBSs were mapped to the promoter sequences of the candidate estrogen responsive ESCC genes in class C2. The genes in class C2 whose promoters contained such cTFBSs were singled out as novel putative estrogen responsive genes in ESCC (class C2A).

To the best of our knowledge our study provided the first computational large-scale analysis of the transcription potential of estrogen responsive ESCC genes and suggests important regulatory potential of these genes. Although we used ESCC as a model, the developed system biology based methodology has a potential to identify hormone responsive genes using other hormone-affected diseases, and provides a framework for identifying hormone responsive genes based on complex diseases.

## Results

### The prediction and identification of putative estrogen responsive genes in ESCC

A sequential two-step process was used to predict and verify estrogen responsive genes in ESCC:

(a) EREs were mapped to the promoters of ESCC genes, and

(b) Based on the experimental evidence the genes in (a) were classified as being estrogen responsive or not.

The 418 ESCC genes were extracted from the Dragon Database of Genes Implicated in Esophageal Cancer (DDEC) [28]. The 1645 putative promoters of these ESCC genes (1200 bp upstream and 200 bp downstream from the transcription start site) were extracted from the Fantom3 CAGE tag data [29] and analyzed for the presence of EREs via the Dragon ERE Finder version 6.0 (http://apps.sanbi.ac.za/ere/index.php) [25]. EREs were mapped to 242 promoter sequences that correspond to 128 ESCC genes. 290 ESCC genes had no EREs mapped to the promoter sequences. Lists of genes that have been experimentally validated to be responsive to estrogen as indicated in the KBERG [26] and ERTargetDB [27] databases were used to confirm which ESCC genes are responsive to estrogen (Additional file 1). Of the 128 genes with predicted EREs, 43.75% (56/128) are known to be estrogen responsive (class C1), while 56.25% (72/128) were new candidate estrogen responsive genes (class C2). EREs did not map to 290 ESCC genes of which 50.34% (146/290) are known to be estrogen responsive (class C3) (Table 1).

### TFBS analysis of estrogen responsive genes in ESCC

**Figure 1:**
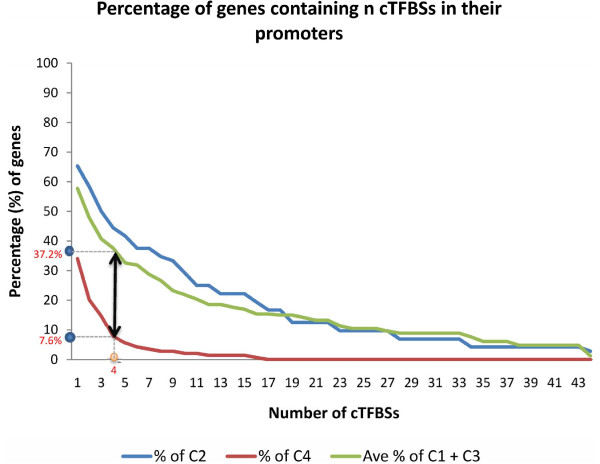
**Defining the minimum number of cTFBSs required for the identification of estrogen responsive ESCC genes.** This figure is a graphical representation of the proportion of genes that contain multiple cTFBSs mapped to their promoters. The graph depicts the threshold defining the maximum difference between the known estrogen responsive genes (C1 and C3) relative to the background set (C4).

TFBS analysis entailed the following three steps: (a) mapping the TFBSs matrix models to the promoters of all ESCC genes, (b) determining the cTFBSs significantly over-represented in class (C1 and C3) relative to class C4 (we determined 44 such cTFBSs), and (c) mapping significantly over-represented cTFBSs determined in (b) to promoters of genes in class C2. In (c), we required that at least four of the 44 cTFBSs map the promoters of each gene in class C2. This threshold corresponds to the maximum difference in the number of genes with these cTFBSs in the positive set (class (C1 and C3)) as compared to the background set (class C4) (Figure 1). All class C2 genes that have such cTFBSs in their promoters (we found 32 such genes) we considered as new candidate estrogen responsive ESCC genes since they have in their promoters both: a/mapped EREs, and b/cTFBSs characteristic of ESCC genes that are responsive to estrogen. This increases confidence that these 32 ESCC genes are responsive to estrogen since due to the similar regulatory potential with estrogen-responsive genes, these genes have higher chance to express when estrogen-responsive genes are expressing and additionally they have ERE that potentially bind ERs.

#### TFBS matrices mapped to the promoters of 418 ESCC genes

The TRANSFAC mammalian matrix models of TFBSs (Tranfac Professional v.11.4) were mapped to the promoters of estrogen responsive genes in ESCC using Match^TM^[30-32]. Of the 522 matrices mapped, 492 mapped to the promoters of the 418 ESCC genes at 165,787 positions, not considering strand (Additional file 2).

#### cTFBSs significantly over-represented in class (C1 and C3) as opposed to class C4

We developed a methodology to identify the cTFBSs significantly over-represented in the known estrogen responsive gene set (class (C1 and C3)) relative to the background set (class C4) (see methodology). Each TFBS was ranked using a method that ensures that the top ranked TFBSs were not only over-represented but also more likely to be co-localized within the promoters. In order to reduce the search space for the potentially significant co-localized TFBSs, a heuristic approach was applied where the 10 TFBSs with the lowest p-value (see Materials and Methods) were selected for subsequent analysis. Every possible combination of cTFBSs that includes some of the 10 TFBS were determined. The significant cTFBSs with a p-value (corrected for multiplicity testing) < 0.05 were selected.

We identified 44 significant cTFBSs consisting of 12 doublet cTFBS, 18 triplet cTFBS, 10 4-element cTFBS, 3 5-element cTFBS and 1 6-element cTFBS (Table 2). The 10 TFBSs that make these cTFBSs are determined by the following TRANSFAC identifiers V$ELK1_01, V$CETS1P54_01, V$YY1_01, V$GATA3_01, V$TAXCREB_02, V$FREAC4_01, V$AREB6_01, V$CREB_Q3, V$E2A_Q6 and V$EBOX_Q6_01. Of the 44 cTFBSs, eight combinations were completely absent in the background set (class C4). The most significant cTFBSs (V$TAXCREB_02, V$AREB6_01, V$CREB_Q3 and V$E2A_Q6) was not present in class C4, but mapped 10 times to the promoters of genes in class C1 and 12 times to the promoters of genes in class C3.

**Table 2:** The cTBFSs mapped to the promoters of the 418 genes differentially expressed in ESCC

**ERE Category**		**C1+C3**		**C1**		**C2**		**C3**		**C4**	
**Number of Genes**		**202**		**56**		**72**		**146**		**144**	
		**Genes**	**%**	**Genes**	**%**	**Genes**	**%**	**Genes**	**%**	**Genes**	**%**
cTFBS38	V$AREB6_01|V$YY1_01|V$CETS1P54_01|V$E2A_Q6	17	8.42%	8	14.29%	9	12.50%	9	6.16%	0	0.00%
cTFBS39	V$AREB6_01|V$FREAC4_01|V$EBOX_Q6_01|V$E2A_Q6	16	7.92%	11	19.64%	7	9.72%	5	3.42%	0	0.00%
cTFBS44	V$TAXCREB_02|V$AREB6_01|V$CETS1P54_01|V$EBOX_Q6_01|V$CREB_Q3|V$E2A_Q6	16	7.92%	9	16.07%	7	9.72%	7	4.79%	0	0.00%
cTFBS43	V$TAXCREB_02|V$AREB6_01|V$EBOX_Q6_01|V$CREB_Q3|V$E2A_Q6	19	9.41%	9	16.07%	11	15.28%	10	6.85%	0	0.00%
cTFBS17	V$TAXCREB_02|V$FREAC4_01|V$E2A_Q6	18	8.91%	10	17.86%	8	11.11%	8	5.48%	0	0.00%
cTFBS34	V$TAXCREB_02|V$AREB6_01|V$CREB_Q3|V$E2A_Q6	22	10.89%	10	17.86%	14	19.44%	12	8.22%	0	0.00%
cTFBS33	V$TAXCREB_02|V$AREB6_01|V$ELK1_01|V$CREB_Q3	18	8.91%	8	14.29%	8	11.11%	10	6.85%	0	0.00%
cTFBS41	V$TAXCREB_02|V$AREB6_01|V$CETS1P54_01|V$CREB_Q3|V$E2A_Q6	17	8.42%	9	16.07%	9	12.50%	8	5.48%	0	0.00%
cTFBS32	V$TAXCREB_02|V$ELK1_01|V$EBOX_Q6_01|V$CREB_Q3	19	9.41%	10	17.86%	8	11.11%	9	6.16%	1	0.69%
cTFBS23	V$AREB6_01|V$ELK1_01|V$CREB_Q3	19	9.41%	8	14.29%	8	11.11%	11	7.53%	1	0.69%
cTFBS35	V$TAXCREB_02|V$AREB6_01|V$EBOX_Q6_01|V$CREB_Q3	21	10.40%	11	19.64%	11	15.28%	10	6.85%	1	0.69%
cTFBS26	V$AREB6_01|V$FREAC4_01|V$E2A_Q6	19	9.41%	13	23.21%	7	9.72%	6	4.11%	1	0.69%
cTFBS37	V$AREB6_01|V$CETS1P54_01|V$CREB_Q3|V$E2A_Q6	19	9.41%	10	17.86%	9	12.50%	9	6.16%	1	0.69%
cTFBS16	V$TAXCREB_02|V$FREAC4_01|V$CETS1P54_01	20	9.90%	14	25.00%	10	13.89%	6	4.11%	1	0.69%
cTFBS36	V$TAXCREB_02|V$EBOX_Q6_01|V$CREB_Q3|V$E2A_Q6	24	11.88%	12	21.43%	12	16.67%	12	8.22%	1	0.69%
cTFBS42	V$TAXCREB_02|V$AREB6_01|V$CETS1P54_01|V$EBOX_Q6_01|V$E2A_Q6	21	10.40%	12	21.43%	12	16.67%	9	6.16%	1	0.69%
cTFBS22	V$FREAC4_01|V$CETS1P54_01|V$EBOX_Q6_01	18	8.91%	13	23.21%	10	13.89%	5	3.42%	1	0.69%
cTFBS40	V$AREB6_01|V$EBOX_Q6_01|V$CREB_Q3|V$E2A_Q6	21	10.40%	10	17.86%	11	15.28%	11	7.53%	1	0.69%
cTFBS21	V$FREAC4_01|V$CETS1P54_01|V$E2A_Q6	18	8.91%	12	21.43%	7	9.72%	6	4.11%	1	0.69%
cTFBS28	V$AREB6_01|V$EBOX_Q6_01|V$CREB_Q3	23	11.39%	12	21.43%	11	15.28%	11	7.53%	2	1.39%
cTFBS20	V$TAXCREB_02|V$EBOX_Q6_01|V$CREB_Q3	27	13.37%	14	25.00%	13	18.06%	13	8.90%	2	1.39%
cTFBS31	V$TAXCREB_02|V$ELK1_01|V$CETS1P54_01|V$EBOX_Q6_01	23	11.39%	14	25.00%	16	22.22%	9	6.16%	2	1.39%
cTFBS27	V$AREB6_01|V$CREB_Q3|V$E2A_Q6	24	11.88%	11	19.64%	14	19.44%	13	8.90%	2	1.39%
cTFBS8	V$FREAC4_01|V$EBOX_Q6_01	22	10.89%	15	26.79%	13	18.06%	7	4.79%	3	2.08%
cTFBS18	V$TAXCREB_02|V$AREB6_01|V$CREB_Q3	27	13.37%	12	21.43%	17	23.61%	15	10.27%	3	2.08%
cTFBS24	V$AREB6_01|V$CETS1P54_01|V$CREB_Q3	24	11.88%	11	19.64%	9	12.50%	13	8.90%	3	2.08%
cTFBS13	V$TAXCREB_02|V$ELK1_01|V$CREB_Q3	28	13.86%	12	21.43%	11	15.28%	16	10.96%	3	2.08%
cTFBS9	V$AREB6_01|V$FREAC4_01	26	12.87%	15	26.79%	11	15.28%	11	7.53%	4	2.78%
cTFBS19	V$TAXCREB_02|V$CREB_Q3|V$E2A_Q6	30	14.85%	14	25.00%	15	20.83%	16	10.96%	4	2.78%
cTFBS7	V$FREAC4_01|V$E2A_Q6	25	12.38%	14	25.00%	9	12.50%	11	7.53%	4	2.78%
cTFBS30	V$EBOX_Q6_01|V$CREB_Q3|V$E2A_Q6	27	13.37%	13	23.21%	13	18.06%	14	9.59%	4	2.78%
cTFBS1	V$ELK1_01|V$CREB_Q3	32	15.84%	12	21.43%	11	15.28%	20	13.70%	5	3.47%
cTFBS6	V$FREAC4_01|V$CETS1P54_01	27	13.37%	17	30.36%	12	16.67%	10	6.85%	5	3.47%
cTFBS15	V$TAXCREB_02|V$CETS1P54_01|V$EBOX_Q6_01	34	16.83%	18	32.14%	21	29.17%	16	10.96%	5	3.47%
cTFBS25	V$AREB6_01|V$CETS1P54_01|V$E2A_Q6	30	14.85%	16	28.57%	16	22.22%	14	9.59%	5	3.47%
cTFBS10	V$AREB6_01|V$CREB_Q3	31	15.35%	13	23.21%	17	23.61%	18	12.33%	6	4.17%
cTFBS14	V$TAXCREB_02|V$CETS1P54_01|V$E2A_Q6	33	16.34%	15	26.79%	19	26.39%	18	12.33%	6	4.17%
cTFBS12	V$EBOX_Q6_01|V$CREB_Q3	32	15.84%	16	28.57%	15	20.83%	16	10.96%	7	4.86%
cTFBS29	V$AREB6_01|V$EBOX_Q6_01|V$E2A_Q6	38	18.81%	16	28.57%	21	29.17%	22	15.07%	8	5.56%
cTFBS2	V$ELK1_01|V$EBOX_Q6_01	36	17.82%	17	30.36%	18	25.00%	19	13.01%	9	6.25%
cTFBS3	V$CETS1P54_01|V$CREB_Q3	38	18.81%	15	26.79%	14	19.44%	23	15.75%	10	6.94%
cTFBS4	V$CETS1P54_01|V$EBOX_Q6_01	41	20.30%	21	37.50%	24	33.33%	20	13.70%	11	7.64%
cTFBS5	V$TAXCREB_02|V$CREB_Q3	43	21.29%	18	32.14%	23	31.94%	25	17.12%	12	8.33%
cTFBS11	V$AREB6_01|V$E2A_Q6	50	24.75%	23	41.07%	25	34.72%	27	18.49%	16	11.11%

44 significant cTFBSs consisting of 12 doublet cTFBS, 18 triplet cTFBS, 10 4-element cTFBS, 3 5-element cTFBS and 1 6-element cTFBS were identified.

#### 44 cTFBSs used to increase confidence in a subset of the new candidate estrogen responsive genes in class C2

**Figure 2:**
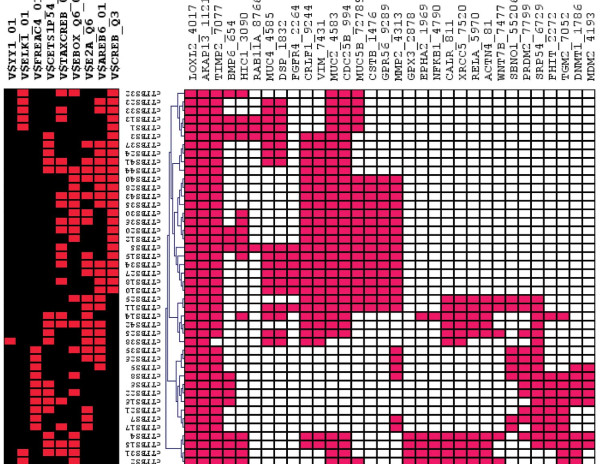
**An overview of the cTFBSs and their regulatory effects on the ESCC genes.** The figure represents a heat map of cTFBSs that are significantly over-represented in the promoters of the known estrogen response genes in ESCC (columns) mapped to the promoters of the new candidate estrogen response genes in C2A (rows). The red shading depicts the association between the gene promoter and the cTFBSs, while white depicts no association.

We mapped the 44 significant cTFBSs to the promoters of the genes in class C1, C2, C3 and C4 thereby generating 574, 567, 561 and 153 predictions of cTFBSs, respectively. This result for the mapping of cTFBSs to the promoters of all categories indicates that multiple cTFBSs are present in the promoters of genes. Moreover, these multiple cTFBSs are more dominant in genes from class (C1 and C3) known to be responsive to estrogen, as well as genes with ERE predictions in their promoters (class C2). Consequently, we applied a threshold that each gene promoter must contain at least four of the significant cTFBSs, as this threshold defines the maximum difference in the number of genes that contain such cTFBSs between the known estrogen responsive gene set (class (C1 and C3)) relative to the background set (class C4) (refer to Figure 1). It was determined that at least four of the significant cTFBSs were present in 51.8% (29/56) of the genes in class C1 (class C1A), 44.4% (32/72) of the genes in class C2 (class C2A), 23.3% (34/146) of the genes in class C3 (class C3A) and 7.6% (11/144) of the genes in class C4 (class C4A) (Additional file 3). An overview of the regulatory effects of the cTFBSs on the 32 genes in class C2A is shown in Figure 2. This figure illustrates each association in class C2A, in the form of a color dot in a heat map format using TMEV [33,34]. The heat map clearly depicts gene clusters based on the cTFBSs common to the promoters of multiple genes in class C2A. 

Moreover, a review of the recently published scientific literature reveals that 47% (15/32) of the C2A genes have now been shown experimentally to be estrogen responsive. These 15 genes include *MUC5B*[35], *MMP2*[36], *LOXL2*[37], *ACTN4*[38], *DNMT1*[39], *GPR56*[40], *MUC4*[40], *WNT7B*[41], *BMP6*[42], *GPX3*[43], *CDC25B*[44], *NFκB1*[45], *PRDM2*[46], *MDM2*[47] and *TIMP2*[48].

## Discussion

In this study, we propose a methodology aimed at providing an insight into the underlying transcription regulatory potential related to genes’ response to estrogen in ESCC. In this systems biology study, we combined information obtained from several databases, genomic sequences of promoters of relevant genes, and analysis of transcription regulation potential of these genes to infer if the genes are estrogen responsive. Two computational components are used to suggest ESCC genes responsive to estrogen: 1) the ERE prediction (made by Dragon ERE Finder version 6.0), and 2) predicted cTFBSs that characterize the promoters of known estrogen responsive ESCC genes (these were obtained based on methodology we developed in this study). These cTFBSs were mapped to the promoters of ESCC genes not being known to be responsive to estrogen, but having ERE predictions in their promoters. In this way we increased the confidence that the ESCC genes with ERE predictions are responsive to estrogen since they, in addition to EREs, also contain cTFBSs characteristic of estrogen responsive ESCC genes.

### ESCC genes predicted to be responsive to estrogen

Carroll et al. has reported that ER binds selectively to a limited number of sites, majority of which are distant from the transcriptional start sites of regulated genes and that direct ER binding requires the presence of forkhead factor (foxa1) binding in close proximity [49]. However, several computational approaches has been undertaken to identify target genes based on the presence of EREs in the proximal promoter regions [25,50]. Bourdeau et al. in particular screened for EREs that were conserved in the human and mouse genome and identified 660 gene proximal EREs of which several were validated as genuine ER interaction sites [50]. This analysis has also been restricted to the proximal promoter region due to computational limitations and regulatory TFs binding closer to the transcription start site. EREs were mapped to the promoters of 418 ESCC genes using the Dragon ERE Finder version 6.0 (http://apps.sanbi.ac.za/ere/index.php). Bajic et al. (2003) have demonstrated that this ERE locator predicts known ERE and estrogen responsive genes at a sensitivity of 0.83. We further identified which of the ESCC genes are known to be responsive to estrogen using the KBERG [26] and ERTargetDB [27] databases. Of the 128 predicted estrogen responsive genes, 43.75% (56/128) are known to be estrogen responsive, while 56.25% (72/128) were novel putative estrogen responsive genes. These 72 genes lay the foundation for increasing insights into the molecular events triggered by estrogen via an ERE dependant mode of regulation in ESCC. EREs did not map to 290 ESCC genes of which 50.34% (146/290) are known to be responsive to estrogen. The promoters of these 146 gene did not contain an ERE motif, but the genes are known to be responsive to estrogen. The response to estrogen of these genes may be through the interactions of ERs with other transcription factors forming complexes that do not require the presence of EREs [51]. It is also possible that the ERE models are not sufficiently good to predict EREs in these promoter regions. Our analysis generated four gene categories (Table 1): class C1 (56 ESCC genes), class C2 (72 ESCC genes), class C3 (146 ESCC genes), and class C4 (144 ESCC genes). We found that the four gene categories had a different number of enriched pathways using Kyoto Encyclopedia of Genes and Genomes (KEGG) (see Methodology and Additional file 4). However, in each category the more general KEGG pathway “Pathways in cancer” (hsa05200) enriched with genes forming the gene sets. Other more specialized and equally important pathways show enrichment with genes forming certain categories. Category 1 genes are highly enriched in the pathways such as “Transcriptional misregulation in cancer” (hsa05202), “Small cell lung cancer” (hsa05222), “Melanoma” (hsa05218). Category 2 genes are highly enriched in the pathways e.g. “p53 signaling pathway” (hsa04115), “Bladder cancer” (hsa05219), “Small cell lung cancer” (hsa05222). Category 3 genes are highly enriched for many pathways, e.g. “Prostate cancer” (hsa05215),“Colorectal cancer” (hsa05210), “Small cell lung cancer” (hsa05222), “Chronic myeloid leukemia” (hsa05220), “Endometrial cancer” (hsa05213), etc. Category 4 genes is additionally highly enriched in the “Bladder cancer” (hsa05219) pathway. These categories were used to identify the cTFBSs that characterize the promoters of the 202 (56 + 146) ESCC gene (from class (C1 and C3)) known to be responsive to estrogen.

### The cTFBSs that characterize the promoters of ESCC genes known to be responsive to estrogen

Since gene expression is driven by the cohesive action of multiple TFs binding to specific TFBSs, common cTFBSs may define co-regulated genes [14,52]. We identified cTFBSs significantly over-represented in the promoters of genes known to be responsive to estrogen (class (C1 and C3)) as compared to the background set (class C4). When comparing the 202 (56 +146) known estrogen responsive genes (class (C1 and C3)) to the background set (class C4), we selected the 10 TFBSs (see Material and Methods) to be used in subsequent analysis. Every possible combination of cTFBSs made of these 10 TFBSs were determined. The significant cTFBSs with a p-value (corrected for multiplicity testing) < 0.05 were selected.

44 significant cTFBSs were identified, eight of which were not present in the background set (class C4). The most significant cTFBS comprised of the following TRANSFAC identifiers: V$TAXCREB_02, V$AREB6_01, V$CREB_Q3 and V$E2A_Q6. The above mentioned cTFBS was not present in class C4, but mapped to the promoters of 14.29% of genes in class C1, 6.16% of genes in class C3, and 12.50% of genes in class C2 (Table 2).

V$AREB6_01 is known to bind AREB6 (also known as ZEB1) [53]; V$TAXCREB_02 binds CREB, deltaCREB and Tax/CREB complex [54,55]; V$CREB_Q3 possibly binds CREB1, CREMalpha, deltaCREB, ATF-1, ATF-2, ATF-3, ATF-4, ATF-a, and ATF-2-xbb4; and V$E2A_Q6 possibly binds E2A, TCF4, TCF12, TFF3, ASCL1, MYF3, MYF4, MYF5, and MYF6. None of the above mentioned TFs has been linked to estrogen, but play a role in the progression of cancer [56-59]. Further details of these TFBSs and their associated TFs can be viewed in Additional file 5.

Even though we have identified cTFBSs that characterize the promoter regions of the known estrogen responsive genes in ESCC, it is unclear whether the TFs that bind the TFBSs function as transcriptional activators or transcriptional repressors in the estrogen responsive ESCC genes [60-62]. Nonetheless, these significant cTFBSs are over-represented in the promoters of known estrogen responsive genes and thus can be used to identify genes that are likely co-regulated with genes responsive to estrogen.

### Identification of candidate estrogen responsive ESCC genes with EREs and cTFBSs mapped to the promoters

The 44 significantly over-represented cTFBSs were used to increase confidence in a subset of the new candidate estrogen responsive genes in class C2. It was determined that at least four of the significant cTFBSs were present in 51.8% (29/56) of the genes in class C1 (class C1A), 44.4% (32/72) of the genes in class C2 (class C2A), 23.3% (34/146) of the genes in class C3 (class C3A) and 7.6% (11/144) of the genes in class C4 (class C4A) ( Additional file 3).

The 44 cTFBSs were determined based on class (C1 and C3), but the findings show that the genes with the cTFBSs are concentrated in class C1 (genes both predicted and confirmed to be responsive to estrogen), since class C1 has 28.5% more genes with a cTFBSs in the promoter sequence as compared to class C3. This result indicates that class C1A gene promoters with EREs also contain distinctive cTFBSs that may define multiple co-regulated genes responsive to estrogen. These co-regulated genes may define estrogen responsive genes that function in an ERE-dependent manner. Thus, the 32 genes with putative EREs in class C2A that have at least four of the cTFBSs may be an additional fraction of these co-regulated genes. These results increase confidence in the new candidate estrogen responsive genes in class C2A since they contain both EREs and cTFBSs characteristic of ESCC genes that are responsive to estrogen.

We found 38 TFs that interact with ER via 137 significant (p-value threshold of <0.05) binding sites using BioGRID [63] and the TRANSFAC [31] databases (Additional file 6), of which at least one binding site is in close proximity to the ERE of the 32 genes identified as estrogen responsive. We additionally found 18 (ESR1, ETS1, FOS, GATA4, HIC1, HIF1A, FOXA1, IRF2, AR, MYC, NFKB1, RARA, RELA, STAT3, NR2F2, TP53, WT1, FOSL1) TFs to be self-regulating. Interestingly, one group [49] has reported that their unbiased sequence interrogation of the genuine chromatin binding sites suggests that direct ER binding requires the presence of foxa1 binding in close proximity, as knockdown of FoxA1 expression blocked the association of ER with the chromatin and estrogen induced gene expression. We do not know if this estrogenic response requirement is restricted to breast cancer cells, but 62.5% of the 32 genes we have identified as estrogen responsive has the ERE in close proximity to the FoxA1 binding site. Further, we provide an overview of the potentially co-regulated gene in class C2A in the form of a heat map (Figure 2). Figure 2 clusters class C2A genes based on the presence of common cTFBSs mapped to the gene promoters. Multiple clusters of genes in the heat map show that different groups of genes have different specific combinations of cTFBSs, making them more likely to be co-regulated. *AKAP13* (Gene ID: 11214), *LOXL2* (Gene ID: 4017), and *TIMP2* (Gene ID: 7077) cluster together and contain the highest number of combinations that are common to their promoters. We further ranked the 32 genes based on the number of cTFBSs present in each promoter (Additional file 7). *AKAP13, LOXL2, TIMP2, CDC25B, MUC2, CRLF1, VIM, MMP2* and *MUC5B* are identified as the top nine ranked genes.

**Figure 3:**
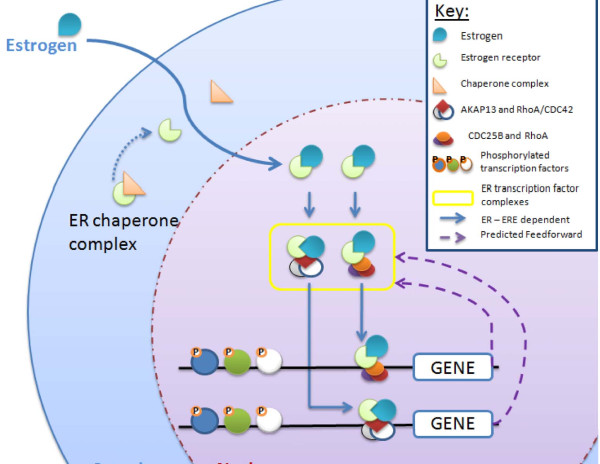
**An illustration of the ERs transcriptional activation process.** This figure is a pictorial representation of the known “estrogen → ER → ERE” dependent transcriptional activation process that requires the candidate estrogen responsive gene products, AKAP13 and CDC25B, as co-factors.

A further literature survey disclosed that *AKAP13* belongs to the Dbl family of proto-oncogenes that function as a Rho family guanine nucleotide exchange factor. It is known to bind and influence the activity of glucocorticoid receptors (GRs) and ERs [64,65]. It has been experimentally demonstrated that AKAP13 interacts with the ligand activated ER to form a tertiary complex with either RhoA or rho related GTPase CDC42 (Figure 3). It has been demonstrated that these complexes bind to ERE sites thereby driving genes expression induced by estrogen [66]. Interestingly, RhoA is also known to be up-regulated in ESCC [67]. Moreover, the p38 MAPK inhibitor SB202190 abrogates ERβ activity by AKAP13 indicating that AKAP13 activates ERβ via the p38 MAPK pathway [66]. Pathway analysis using DAVID [68] indicates that four of our putative estrogen response genes (*FGFR4, RELA, NFκβ* and *CDC25B*) are involved in the MAPK signaling pathway. *CDC25B* belongs to the CDC25 family of phosphatases that activates cyclin dependent kinases by removal of inhibitory phosphates. This gene is also known to bind and influence the activity of nuclear receptors such as progesterone receptor (PR) and ER. It has been experimentally demonstrated that CDC25B interacts with the ligand activated ER in a hormone-dependent ER transactivation manner. Also, the p300/CBP-associated factor and CREB binding protein were shown to interact and synergize with CDC25B and further enhance its co-activation activity [69]. These findings link *AKAP13* and *CDC25B*, two of the top 10 ranked putative estrogen response genes, to estrogen activity and highlight their functioning as co-factors in the ERs transcriptional activity. Because these genes are putative estrogen responsive genes, this finding may be indicative of a cascading event that may be an important step in regulating hormone-dependent ER transactivation. 

Recent publications show that *MUC5B*[35], *MMP2*[36], *LOXL2*[37], *ACTN4*[38], *DNMT1*[39], *GPR56*[40], *MUC4*[40], *WNT7B*[41], *BMP6*[42], *GPX3*[43], *CDC25B*[44], *NFκB1*[45], *PRDM2*[46], *MDM2*[47] and *TIMP2*[48] are responsive to estrogen. These findings further increase confidence that the 32 new candidate estrogen responsive ESCC genes may indeed be estrogen responsive.

## Conclusion

Our study proposes a methodology that provides insight into the regulatory potential of estrogen responsive genes and identifies 32 new candidate estrogen responsive genes using ESCC as the framework. *AKAP13, LOXL2, TIMP2, CDC25B, MUC2, CRLF1, VIM, MMP2* and *MUC5B* were identified as the top nine ranked genes, of which AKAP13 [64,66] and CDC25B [69] have independently been identified in other studies as essential components of ER complexes that are required to drive estrogen induced gene expression. Moreover, estrogen responsiveness of 47% (15 out of 32) of genes predicted by our method is supported by experimental findings in recent publications. These insights into the transcription regulation potential associated with estrogen response provide information of potential interest to those with interest in studying estrogen effects in ESCC and in design estrogen-based EC therapies. This study is the first to use a cancer disease model as the framework to identify hormone responsive genes. Although we used ESCC and estrogen for this purpose, the methodology, however, can be extended analogously to use other diseases as the model and other hormones.

## Methods

### Extracting promoter regions of genes differentially expressed in ESCC

A total of 418 genes were extracted from the Dragon Database of Genes Implicated in Esophageal Cancer (DDEC) [28]. The promoters (1200 bp upstream and 200 bp downstream from the transcription start site, TSS) of all 418 ESCC genes under study were extracted from the Fantom3 CAGE tag data that correspond to 1645 transcription start sites (TSSs) that each have at least five tags in the tag cluster and a minimum of three tags corresponding to the representative tag [29].

### Annotating and classifying ESCC genes according to predicted and validated estrogen response

Dragon ERE Finder version 6.0 (http://apps.sanbi.ac.za/ere/index.php) was used to predict EREs in the promoter regions of ESCC genes. A sensitivity of 0.83 was used as recommended in [25]. Based on the presence of predicted EREs the 418 ESCC genes were divided into two groups: 1) genes whose promoters contain predicted EREs, and 2) genes lacking predicted EREs. These two gene groups were further divided into those known to be experimentally confirmed as estrogen responsive and those that are not, by cross-checking the all ESCC genes against the estrogen responsive genes in the KBERG [26] and ERtargetDB [26] databases. The KBERG database contained 1516 experimentally confirmed estrogen-responsive genes. The ERTargetDB, database contained: (a) 40 genes with 48 experimentally verified ERE direct binding sites and 11 experimentally verified ERE tethering sites; (b) 42 genes identified via ChIP-on-chip assay for estrogen binding and (c) 355 genes from gene expression microarrays, all of which were included in this study. However, this study excludes the 2659 computationally predicted estrogen responsive genes included the ERTargetDB, database.

Thus we classified the 418 ESCC genes into the following four categories:

C1/ESCC genes with predicted EREs in their promoters and known as estrogen responsive,

C2/ESCC genes with predicted EREs in their promoters but not known as estrogen responsive,

C3/ESCC genes having no predicted EREs in their promoters, but known as estrogen responsive,

C4/ESCC genes having no predicted EREs in their promoters and not known as estrogen responsive.

We used these categories to develop a methodology for the identification of sets of co-localized TFBSs (cTFBSs) that characterize the promoters of the known estrogen responsive gene set (class C1 and C3) as opposed to the background set (class C4).

### Gene-set pathway enrichment analysis

Gene enrichment in Kyoto Encyclopedia of Genes and Genomes (KEGG) pathways was calculated using the Fisher’s exact test based the hypergeometric distribution [70] with all genes that are associated to at least one KEGG pathway. All other genes were discarded for the analysis. The set of genes was compared to the set of all human genes that have at least one KEGG pathway associated. Finally all p-values were adjusted using the method by Benjamini and Hochberg to control the false discovery rate [71] and only pathways retained were the adjusted p-value is below 0.01. In total 253 KEGG pathways were under consideration.

### Identification of cTFBSs

TRANSFAC mammalian matrix profiles of TFBSs were mapped to the promoters of all 418 ESCC genes under study by using Match^TM^[30-32] with minFP profiles. We developed the following 3-step methodology to identify the cTFBSs significantly over-represented in the known estrogen responsive genes (class C1 and C3) as opposed to the background set (class C4):

1. Given the full set of 522 TRANSFAC mammalian matrices, we calculated the p-value for any given matrix pair M_i_M_j_ being present in greater proportions in class (C1 and C3) promoters as opposed to class C4. We did not take strand into account. The p-values were calculated using the one-sided Fisher’s exact test. In the case where Mi = Mj, we corrected the p-values for multiple testing by a factor of 522 (Bonferroni); when Mi ≠ Mj, we corrected by a factor of 522^2^-522/2.

2. Having calculated the corrected p-value for each M_i_M_j_ pair, we scored each individual matrix M_i_ by *Si* = ∑ _*j* = 1_^*S*22^*p*(*MiMj*). Roughly, one would expect to have more abundant M_i_ in class (C1 and C3) promoters as opposed to class C4 promoters when the smaller the score S_i_. Additionally, groups of matrices with similarly low scores tend to co-localize more often in the promoters of class (C1 and C3) than in the promoters of class C4 genes.

3. We selected 10 matrices with the lowest p-values, calculated as described above. Using these 10 matrices we tested for the disproportionate presence of all combinations consisting of 2 to 10 of these matrices (cTFBSs) between the class (C1 and C3) and class C4 gene promoter sets. A Bonferroni correction factor of (_*k*_^*n*^) was applied, where n = 10 and k equates to the number of matrices under consideration per combination. Significance was determined at the corrected p-value ≤ 0.05.

In the above manner a total of 44 cTFBSs were found to be significantly over-represented in the promoters of class (C1 and C3).

### Annotation of class C2 genes implicated in ESCC as estrogen responsive

We found that many of the 44 over-represented cTFBSs were indeed present in class C2. However, we applied a threshold that each gene must map at least four of the significant cTFBSs, as this threshold defines the maximum difference between the known estrogen responsive gene set (class (C1 and C3)) relative to the background set (class C4). Thus, by using four cTFBSs as a threshold, we putatively annotated 44.4% of the genes in as being estrogen responsive. These annotations are made viewable in the form of a heat-map using TMEV [33,34]. The heat map is based on hierarchical clustering with average linkage and Euclidian distance. The shade of red depicts an association between the gene and the cTFBSs, while no shade indicates that the cTFBS could not be mapped onto the gene’s promoter.

## Competing interests

The authors declare that they have no competing interests.

## Authors’ contributions

ME, CM, and VBB conceptualized the study and wrote the manuscript. ME, CM and SS collected and integrated the data and performed the analysis. All authors read and approved the final manuscript.

## Supplementary Material

Additional file 1 **ESCC genes categorized based on ERE predictions and experimental evidence of estrogen responsiveness.** Appendix I.xls contains all the 418 ESCC genes tabulated based on ERE predictions and experimental evidence of estrogen responsiveness. Click here for file

Additional file 2 **TFBS matrices mapped to the promoters of the ESCC genes.** Appendix II.xls contains all the TFBS matrices mapped to the promoters of the ESCC genes. Click here for file

Additional file 3 **Multiple cTFBSs mapped to the promoters of the ESCC genes.** Appendix III.xls contains the number of multiple cTFBS matrices mapped to the promoters of the ESCC genes in categories C1, C2, C3 nad C4. Click here for file

Additional file 4 **Pathways enriched in the Four ESCC gene categories. **Appendix IV.xls contains details of the enriched KEGG pathways in the four gene categories. Click here for file

Additional file 5 **The most significant cTFBS. **Appendix V.xls contains details of the most significant combination of cTFBSs comprising V$TAXCREB_02, V$AREB6_01, V$CREB_Q3 and V$E2A_Q6. Click here for file

Additional file 6 **The significant TFBSs and their associated TFs.** Appendix VI.xls contains details of the 137 significant TFBSs and the TFs that bind them. Click here for file

Additional file 7 **Ranked list of putative estrogen responsive ESCC genes.** Appendix VII.xls contains a ranked list of putative estrogen response ESCC genes based on the number of cTFBSs mapped the promoter. Click here for file
