# Supplementary material for: Identification of estrogen responsive genes using esophageal squamous cell carcinoma (ESCC) as a model
Source: BMC Syst Biol. 2012 Oct 26;6:135. doi: 10.1186/1752-0509-6-135 (PMC3495646; doi:10.1186/1752-0509-6-135)
Supplement: Additional file 5 — The most significant cTFBS. Appendix V.xls contains details of the most significant combination of cTFBSs comprising V$TAXCREB_02, V$AREB6_01, V$CREB_Q3 and V$E2A_Q6. [file 1752-0509-6-135-S5.docx]

**Appendix IV: Details of the most significant combination of cTFBSs comprising V$TAXCREB_02, V$AREB6_01, V$CREB_Q3 and V$E2A_Q6.**

M00412 (V$AREB6_01) is known to bind AREB6 (also known as ZEB1) [1]. ZEB1 has been implicated in the transcriptional repression of genes such as IL2 [2], CDH13 (found in C3) [3], CDH1 (found in C2) [4], ARHGAP24 [5], PKP3 [6] and BZLF1 [7]. ZEB1 and SIP1 are also implicated in the transcriptional repression of miR-200b [8] and conversely, the expression levels of ZEB1 is regulated by the microRNA-200 family (miR-200a, miR-200b, miR-200c, miR-141 and miR-429) and miR-205 [9] thereby producing a double negative feedback loop. Published biological data demonstrate that the miR-200 family plays a major role in specifying the epithelial phenotype by preventing expression of the transcription repressors, ZEB1/deltaEF1 and SIP1/ZEB2 [8, 9]. This epithelial to mesenchymal transition (EMT) occurs during embryologic development to allow tissue remodeling and is proposed to be a key step in the metastasis of epithelial-derived tumors. Thus, the double negative feedback loop regulates cellular phenotype and has direct relevance to the role of these factors in tumor progression. ZEB1 has also been found to mediate disintegration of intercellular adhesion and EMT via the transcriptional repression of genes such as CRB3, LLGL2 and MPP5 [10]. Thus, it has been documented as a key biomarker of aggressive cancer at high risk of recurrence and is recognized as a therapeutic target [11].

M00115 (V$TAXCREB_02) binds CREB, deltaCREB and Tax/CREB complex [12, 13]. Over-expression of CREB has been linked to numerous cancer types such as ovarian cancer [14], gastrointestinal cancer [15], breast cancer [16], duodenal cancer [17] and prostate cancer [5]. CREB transcribes more than 5000 target genes. Expression of CREB is directly regulated by miR-34b and the miR-34b promoter was shown to be methylated in the leukemia cell lines used [18]. CREB has been documented as a prognostic marker in acute myeloid leukemia [12].

The PWMs designated M00801 (V$CREB_Q3) and M00973 (V$E2A_Q6) are motifs known to bind multiple TFs, as these matrices were derived from a collection of TFBSs associated with the binding of different TFs [19]. Thus, the TF associated with these matrices cannot be defined.

M00801 (V$CREB_Q3) possibly binds CREB1, CREMalpha, deltaCREB, ATF-1, ATF-2, ATF-3, ATF-4, ATF-a, and ATF-2-xbb4. Tax represses the transcription of TATA-less CCNA2, CCND3 and POLA1 promoters by attaching to the CREB/ATF complex bound to the CREB/ATF binding site [20]. The interaction between Tax and CREB is highly specific, whereas Tax interacts with ATF only marginally despite the extensive sequence similarities between CREB and ATF. Thus, if CREB is not bound to the motif then transcriptional repression is absent [21]. ATF-1 and ATF-2 is up-regulated and bind to Tax-responsive elements in infected bovine B lymphocytes [22]. Additionally, BATF is up-regulated in human B lymphocytes infected by Epstein-Barr virus (EBV) and was shown to negatively impact the expression of a *BZLF1* reporter gene and to reduce the frequency of lytic replication in latently infected cells [23], suggesting that BATF impact viral and cellular gene expression by promote viral latency and control lytic-cycle entry. ZEB1, CREB1 and ATF1 are members of the bZIP family of leucine-zipper transactivators [7, 24].

M00973 (V$E2A_Q6) possibly binds E2A, TCF4, TCF12, TFF3, ASCL1, MYF3, MYF4, MYF5, and MYF6. These nuclear proteins belong to the basic helix-loop-helix family of transcription factors, except TFF3. Dysregulation of the E2A/TCF12 proteins have been implicated in t(8;21) leukemogenesis [25]. The ASCL1 protein was shown to be expressed in neuroendocrine cells of normal prostatic gland and absent in prostate cancers without neuroendocrine differentiation, whereas ASCL1 was expressed in 25% of prostate cancers with neuroendocrine differentiation/untreated [26]. MYF3, MYF4, MYF5, and MYF6 were shown to be activators of muscle differentiation and induces the expression of cell cycle regulators, p21, Rb, and cyclin D3 [27]. TFF3 was shown to be commonly expressed in hepatocellular carcinoma [28], while follicular neoplastic lesions of the thyroid gland have decreased expression of TFF3 compared to normal thyroid tissue [29].

**Reference**

1. Ikeda K, Kawakami K: **DNA binding through distinct domains of zinc-finger-homeodomain protein AREB6 has different effects on gene transcription**. *Eur J Biochem* 1995, **233**(1):73-82.

2. Wang J, Lee S, Teh CE, Bunting K, Ma L, Shannon MF: **The transcription repressor, ZEB1, cooperates with CtBP2 and HDAC1 to suppress IL-2 gene activation in T cells**. *Int Immunol* 2009, **21**(3):227-235.

3. Adachi Y, Takeuchi T, Nagayama T, Ohtsuki Y, Furihata M: **Zeb1-mediated T-cadherin repression increases the invasive potential of gallbladder cancer**. *FEBS Lett* 2009, **583**(2):430-436.

4. Huang W, Zhang Y, Varambally S, Chinnaiyan AM, Banerjee M, Merajver SD, Kleer CG: **Inhibition of CCN6 (Wnt-1-induced signaling protein 3) down-regulates E-cadherin in the breast epithelium through induction of snail and ZEB1**. *Am J Pathol* 2008, **172**(4):893-904.

5. Dominguez G, Pena C, Silva J, Garcia JM, Garcia V, Rodriguez R, Cantos B, Citores MJ, Espana P, Bonilla F: **The presence of an intronic deletion in p73 and high levels of ZEB1 alter the TAp73/DeltaTAp73 ratio in colorectal carcinomas**. *J Pathol* 2006, **210**(4):390-397.

6. Aigner K, Descovich L, Mikula M, Sultan A, Dampier B, Bonne S, van Roy F, Mikulits W, Schreiber M, Brabletz T *et al*: **The transcription factor ZEB1 (deltaEF1) represses Plakophilin 3 during human cancer progression**. *FEBS Lett* 2007, **581**(8):1617-1624.

7. Kraus RJ, Perrigoue JG, Mertz JE: **ZEB negatively regulates the lytic-switch BZLF1 gene promoter of Epstein-Barr virus**. *J Virol* 2003, **77**(1):199-207.

8. Bracken CP, Gregory PA, Kolesnikoff N, Bert AG, Wang J, Shannon MF, Goodall GJ: **A double-negative feedback loop between ZEB1-SIP1 and the microRNA-200 family regulates epithelial-mesenchymal transition**. *Cancer Res* 2008, **68**(19):7846-7854.

9. Gregory PA, Bert AG, Paterson EL, Barry SC, Tsykin A, Farshid G, Vadas MA, Khew-Goodall Y, Goodall GJ: **The miR-200 family and miR-205 regulate epithelial to mesenchymal transition by targeting ZEB1 and SIP1**. *Nat Cell Biol* 2008, **10**(5):593-601.

10. Aigner K, Dampier B, Descovich L, Mikula M, Sultan A, Schreiber M, Mikulits W, Brabletz T, Strand D, Obrist P *et al*: **The transcription factor ZEB1 (deltaEF1) promotes tumour cell dedifferentiation by repressing master regulators of epithelial polarity**. *Oncogene* 2007, **26**(49):6979-6988.

11. Singh M, Spoelstra NS, Jean A, Howe E, Torkko KC, Clark HR, Darling DS, Shroyer KR, Horwitz KB, Broaddus RR *et al*: **ZEB1 expression in type I vs type II endometrial cancers: a marker of aggressive disease**. *Mod Pathol* 2008, **21**(7):912-923.

12. Cheng L, Li L, Qiao X, Liu J, Yao X: **Functional characterization of the promoter of human kinetochore protein HEC1: novel link between regulation of the cell cycle protein and CREB family transcription factors**. *Biochim Biophys Acta* 2007, **1769**(9-10):593-602.

13. Paca-Uccaralertkun S, Zhao LJ, Adya N, Cross JV, Cullen BR, Boros IM, Giam CZ: **In vitro selection of DNA elements highly responsive to the human T-cell lymphotropic virus type I transcriptional activator, Tax**. *Mol Cell Biol* 1994, **14**(1):456-462.

14. Linnerth NM, Greenaway JB, Petrik JJ, Moorehead RA: **cAMP response element-binding protein is expressed at high levels in human ovarian adenocarcinoma and regulates ovarian tumor cell proliferation**. *Int J Gynecol Cancer* 2008, **18**(6):1248-1257.

15. Chinnappan D, Qu X, Xiao D, Ratnasari A, Weber HC: **Human gastrin-releasing peptide receptor gene regulation requires transcription factor binding at two distinct CRE sites**. *Am J Physiol Gastrointest Liver Physiol* 2008, **295**(1):G153-G162.

16. Chhabra A, Fernando H, Watkins G, Mansel RE, Jiang WG: **Expression of transcription factor CREB1 in human breast cancer and its correlation with prognosis**. *Oncol Rep* 2007, **18**(4):953-958.

17. Qu X, Xiao D, Weber HC: **Human gastrin-releasing peptide receptor mediates sustained CREB phosphorylation and transactivation in HuTu 80 duodenal cancer cells**. *FEBS Lett* 2002, **527**(1-3):109-113.

18. Pigazzi M, Manara E, Baron E, Basso G: **miR-34b targets cyclic AMP-responsive element binding protein in acute myeloid leukemia**. *Cancer Res* 2009, **69**(6):2471-2478.

19. Matys V, Kel-Margoulis OV, Fricke E, Liebich I, Land S, Barre-Dirrie A, Reuter I, Chekmenev D, Krull M, Hornischer K *et al*: **TRANSFAC and its module TRANSCompel: transcriptional gene regulation in eukaryotes**. *Nucleic Acids Res* 2006, **34**(Database issue):D108-110.

20. Kibler KV, Jeang KT: **CREB/ATF-dependent repression of cyclin a by human T-cell leukemia virus type 1 Tax protein**. *J Virol* 2001, **75**(5):2161-2173.

21. Adya N, Zhao LJ, Huang W, Boros I, Giam CZ: **Expansion of CREB's DNA recognition specificity by Tax results from interaction with Ala-Ala-Arg at positions 282-284 near the conserved DNA-binding domain of CREB**. *Proc Natl Acad Sci U S A* 1994, **91**(12):5642-5646.

22. Adam E, Kerkhofs P, Mammerickx M, Burny A, Kettmann R, Willems L: **The CREB, ATF-1, and ATF-2 transcription factors from bovine leukemia virus-infected B lymphocytes activate viral expression**. *J Virol* 1996, **70**(3):1990-1999.

23. Johansen LM, Deppmann CD, Erickson KD, Coffin WF, 3rd, Thornton TM, Humphrey SE, Martin JM, Taparowsky EJ: **EBNA2 and activated Notch induce expression of BATF**. *J Virol* 2003, **77**(10):6029-6040.

24. Shaywitz AJ, Greenberg ME: **CREB: a stimulus-induced transcription factor activated by a diverse array of extracellular signals**. *Annu Rev Biochem* 1999, **68**:821-861.

25. Zhang J, Kalkum M, Yamamura S, Chait BT, Roeder RG: **E protein silencing by the leukemogenic AML1-ETO fusion protein**. *Science* 2004, **305**(5688):1286-1289.

26. Rapa I, Ceppi P, Bollito E, Rosas R, Cappia S, Bacillo E, Porpiglia F, Berruti A, Papotti M, Volante M: **Human ASH1 expression in prostate cancer with neuroendocrine differentiation**. *Mod Pathol* 2008, **21**(6):700-707.

27. Roy K, de la Serna IL, Imbalzano AN: **The myogenic basic helix-loop-helix family of transcription factors shows similar requirements for SWI/SNF chromatin remodeling enzymes during muscle differentiation in culture**. *J Biol Chem* 2002, **277**(37):33818-33824.

28. Khoury T, Chadha K, Javle M, Donohue K, Levea C, Iyer R, Okada H, Nagase H, Tan D: **Expression of intestinal trefoil factor (TFF-3) in hepatocellular carcinoma**. *Int J Gastrointest Cancer* 2005, **35**(3):171-177.

29. Patel MR, Bryson PC, Shores CG, Hart CF, Thorne LB, Deal AM, Zanation AM: **Trefoil factor 3 immunohistochemical characterization of follicular thyroid lesions from tissue microarray**. *Arch Otolaryngol Head Neck Surg* 2009, **135**(6):590-596.
